# Supplementary material for: HIVprotI: an integrated web based platform for prediction and design of HIV proteins inhibitors
Source: J Cheminform. 2018 Mar 9;10:12. doi: 10.1186/s13321-018-0266-y (PMC5845081; doi:10.1186/s13321-018-0266-y)
Supplement: Supplementary file 2 — Additional file 2. Source code of HIVProtI web server. [file 13321_2018_266_MOESM2_ESM.zip › HIVprotI_Source-code/hivprotI/weka-3-6-6/doc/weka/datagenerators/ClusterGenerator.html]

ClusterGenerator


---


|  |  |  |
| --- | --- | --- |
| **Overview** \| **Package** \| **Class** \| **Tree** \| **Deprecated** \| **Index** \| **Help** \| **Weka's home** | |  |
| **PREV CLASS**   **NEXT CLASS** | **FRAMES**    **NO FRAMES**     **All Classes** |  |
| SUMMARY: NESTED \| FIELD \| CONSTR \| METHOD | DETAIL: FIELD \| CONSTR \| METHOD |  |


---


## weka.datagenerators Class ClusterGenerator

```
java.lang.Object
  weka.datagenerators.DataGenerator
      weka.datagenerators.ClusterGenerator
```

**All Implemented Interfaces:**: java.io.Serializable, OptionHandler, Randomizable, RevisionHandler

**Direct Known Subclasses:**: BIRCHCluster, SubspaceCluster

---

``` public abstract class ClusterGenerator extends DataGenerator ```

Abstract class for cluster data generators.

Example usage as the main of a datagenerator called RandomGenerator:

```
 public static void main(String[] args) {
   try {
     DataGenerator.makeData(new RandomGenerator(), args);
   } 
   catch (Exception e) {
     e.printStackTrace();
     System.err.println(e.getMessage());
   }
 }
```

**Version:**
:   $Revision: 1.6 $

**Author:**
:   Gabi Schmidberger (gabi@cs.waikato.ac.nz), FracPete (fracpete at waikato dot ac dot nz)

**See Also:**: Serialized Form

---

**Constructor Summary**

`ClusterGenerator()`
  
          initializes the generator


| **Method Summary** | |
| --- | --- |
| `java.lang.String` | `booleanColsTipText()`             Returns the tip text for this property |
| `java.lang.String` | `classFlagTipText()`             Returns the tip text for this property |
| `Range` | `getBooleanCols()`             returns the range of boolean attributes. |
| `boolean` | `getClassFlag()`             Gets the class flag. |
| `Range` | `getNominalCols()`             returns the range of nominal attributes |
| `int` | `getNumAttributes()`             Gets the number of attributes that should be produced. |
| `java.lang.String[]` | `getOptions()`             Gets the current settings of the classifier. |
| `java.util.Enumeration` | `listOptions()`             Returns an enumeration describing the available options. |
| `java.lang.String` | `nominalColsTipText()`             Returns the tip text for this property |
| `java.lang.String` | `numAttributesTipText()`             Returns the tip text for this property |
| `void` | `setBooleanCols(Range value)`             Sets which attributes are boolean. |
| `void` | `setBooleanIndices(java.lang.String rangeList)`             Sets which attributes are boolean |
| `void` | `setClassFlag(boolean classFlag)`             Sets the class flag, if class flag is set, the cluster is listed as class atrribute in an extra attribute. |
| `void` | `setNominalCols(Range value)`             Sets which attributes are nominal. |
| `void` | `setNominalIndices(java.lang.String rangeList)`             Sets which attributes are nominal |
| `void` | `setNumAttributes(int numAttributes)`             Sets the number of attributes the dataset should have. |
| `void` | `setOptions(java.lang.String[] options)`             Sets the options. |

**Methods inherited from class weka.datagenerators.DataGenerator**

`debugTipText, defaultOutput, defineDataFormat, formatTipText, generateExample, generateExamples, generateFinished, generateStart, getDatasetFormat, getDebug, getNumExamplesAct, getOutput, getRandom, getRelationName, getSeed, getSingleModeFlag, makeData, outputTipText, randomTipText, relationNameTipText, seedTipText, setDatasetFormat, setDebug, setOutput, setRandom, setRelationName, setSeed`

**Methods inherited from class java.lang.Object**

`equals, getClass, hashCode, notify, notifyAll, toString, wait, wait, wait`

**Methods inherited from interface weka.core.RevisionHandler**

`getRevision`

**Constructor Detail**

### ClusterGenerator

```
public ClusterGenerator()
```

:   initializes the generator


**Method Detail**

### listOptions

```
public java.util.Enumeration listOptions()
```

:   Returns an enumeration describing the available options.

    :   **Specified by:**: `listOptions` in interface `OptionHandler` **Overrides:**: `listOptions` in class `DataGenerator`
    :   **Returns:**: an enumeration of all the available options.

---


### setOptions

```
public void setOptions(java.lang.String[] options)
                throws java.lang.Exception
```

:   Sets the options.

    :   **Specified by:**: `setOptions` in interface `OptionHandler` **Overrides:**: `setOptions` in class `DataGenerator`
    :   **Parameters:**: `options` - the options **Throws:**: `java.lang.Exception` - if invalid option

---


### getOptions

```
public java.lang.String[] getOptions()
```

:   Gets the current settings of the classifier.

    :   **Specified by:**: `getOptions` in interface `OptionHandler` **Overrides:**: `getOptions` in class `DataGenerator`
    :   **Returns:**: an array of strings suitable for passing to setOptions **See Also:**: `DataGenerator.removeBlacklist(String[])`

---


### setNumAttributes

```
public void setNumAttributes(int numAttributes)
```

:   Sets the number of attributes the dataset should have.

    :   **Parameters:**: `numAttributes` - the new number of attributes

---


### getNumAttributes

```
public int getNumAttributes()
```

:   Gets the number of attributes that should be produced.

    :   **Returns:**: the number of attributes that should be produced

---


### numAttributesTipText

```
public java.lang.String numAttributesTipText()
```

:   Returns the tip text for this property

    :   **Returns:**: tip text for this property suitable for displaying in the explorer/experimenter gui

---


### setClassFlag

```
public void setClassFlag(boolean classFlag)
```

:   Sets the class flag, if class flag is set,
    the cluster is listed as class atrribute in an extra attribute.

    :   **Parameters:**: `classFlag` - the new class flag

---


### getClassFlag

```
public boolean getClassFlag()
```

:   Gets the class flag.

    :   **Returns:**: the class flag

---


### classFlagTipText

```
public java.lang.String classFlagTipText()
```

:   Returns the tip text for this property

    :   **Returns:**: tip text for this property suitable for displaying in the explorer/experimenter gui

---


### setBooleanIndices

```
public void setBooleanIndices(java.lang.String rangeList)
```

:   Sets which attributes are boolean

    :   **Parameters:**: `rangeList` - a string representing the list of attributes. Since the string will typically come from a user, attributes are indexed from 1.

---


### setBooleanCols

```
public void setBooleanCols(Range value)
```

:   Sets which attributes are boolean.

    :   **Parameters:**: `value` - the range to use

---


### getBooleanCols

```
public Range getBooleanCols()
```

:   returns the range of boolean attributes.

    :   **Returns:**: the range of boolean attributes

---


### booleanColsTipText

```
public java.lang.String booleanColsTipText()
```

:   Returns the tip text for this property

    :   **Returns:**: tip text for this property suitable for displaying in the explorer/experimenter gui

---


### setNominalIndices

```
public void setNominalIndices(java.lang.String rangeList)
```

:   Sets which attributes are nominal

    :   **Parameters:**: `rangeList` - a string representing the list of attributes. Since the string will typically come from a user, attributes are indexed from 1.

---


### setNominalCols

```
public void setNominalCols(Range value)
```

:   Sets which attributes are nominal.

    :   **Parameters:**: `value` - the range to use

---


### getNominalCols

```
public Range getNominalCols()
```

:   returns the range of nominal attributes

    :   **Returns:**: the range of nominal attributes

---


### nominalColsTipText

```
public java.lang.String nominalColsTipText()
```

:   Returns the tip text for this property

    :   **Returns:**: tip text for this property suitable for displaying in the explorer/experimenter gui


---


|  |  |  |
| --- | --- | --- |
| **Overview** \| **Package** \| **Class** \| **Tree** \| **Deprecated** \| **Index** \| **Help** \| **Weka's home** | |  |
| **PREV CLASS**   **NEXT CLASS** | **FRAMES**    **NO FRAMES**     **All Classes** |  |
| SUMMARY: NESTED \| FIELD \| CONSTR \| METHOD | DETAIL: FIELD \| CONSTR \| METHOD |  |


---
